# A scoping review of the evidence available for the use of salons as health promotion environments, for the prevention and management of non-communicable diseases in women from different ethnic backgrounds

**DOI:** 10.3389/fpubh.2023.1161645

**Published:** 2023-07-17

**Authors:** Prerana Kaneri, Marjorie Lima do Vale, Seeromanie Harding, Mariam Molokhia

**Affiliations:** Department of Population Health Sciences, School of Life Course and Population Sciences, King’s College London, London, United Kingdom

**Keywords:** salon, ethnicity, community partnership model, health inequalities, review

## Abstract

**Introduction:**

Women from different ethnic backgrounds are disproportionately affected by non-communicable diseases (NCDs). Underpinned by the community capital they harness, hairdressers have successfully delivered NCD prevention programmes, particularly for African-American women in disadvantaged areas. Integrating community organisations and networks into existing primary care pathways can provide a sustainable process to address inequalities in access to health care. This scoping review aimed to map the evidence about interventions based in beauty salons, particularly formative research phases, including co-development, community participation, theoretical or conceptual underpinnings, as well as aspects related to training and incentivisation of salon staff, evaluation and equity.

**Methods:**

The methodological framework was based on the seminal guidance of Arksey and O’Malley, using the ‘PCC’ (participants, concept, context) structure with incorporation of other relevant materials. Studies eligible for inclusion were salon-based health interventions (concept) focused on NCDs prevention (context), targeting women (participants) from different ethnic backgrounds and published in English. The searches were conducted across PubMed, Web of Science and OVID in June 2020 and updated in January 2023, with reference lists also screened. The Reach, Effectiveness, Adoption, Implementation, and Maintenance RE-AIM framework was used to explore the potential public health impact.

**Results:**

419 titles and abstracts were screened, with eight (2%) meeting the inclusion criteria, all based in the United States of America. Two used formative phases to inform intervention development, three described evidence of co-development with key stakeholders or experts within the community and five studies referred to theoretical or conceptual frameworks. Incentivisation was provided to salon staff in five of the studies, and to clients in three of the studies. Four of the investigations collated data on socioeconomic characteristics of the target population.

**Discussion:**

Formative research in the scoped studies was weakly reported upon. Community participation was implicit in each of the scoped studies, yet its application varied considerably. Theoretical and conceptual frameworks were not consistently used, and there was inadequate process evaluation to ensure equitable reach and retention of targeted groups, suggesting a more concerted effort to address health equity is needed for future interventions.

## Introduction

There has been a progressive shift in attention to the intersection of ethnicity, socio-economic circumstances (SEC) and health. We use the term ‘ethnic minority’ groups in the text to reflect other terms such as ‘minority ethnic group’ or ‘Black and Asian’ groups with the understanding that they both reflect the socio-economic, cultural and political intersections that drive ethnic inequalities in health (1). In England, people from White ethnic groups (i.e., White British) are least likely to live in the 10% most income deprived neighbourhoods compared to all other ethnic groups. Available evidence also indicates that certain ethnic minority groups have increased susceptibility to specific Non-Communicable Diseases (NCDs) and experience poorer health outcomes. Such disparities can be seen in the age of diagnosis of some cancers, with the median age for diagnosis of breast cancer in Black British women being 50 years, compared to 62 years for White British women (2). Individuals from ethnic minority groups have also been shown to have poor outcomes from asthma globally, despite experiencing a relatively low risk of developing asthma (3, 4). Additionally, gender inequalities exists in asthma, (5) and cardiovascular risk, which is not routinely assessed in young women, resulting in undiagnosed morbidity and premature mortality (6). Excess mortality (relative to gender and age-specific rates for all born in England and Wales) can be observed for several migrant groups and in socioeconomically deprived groups. Health promotion strategies should address health risks from deprivation as well as underlying disease susceptibility in ethnic minority groups (7).

Community partnerships can support creation of innovative health services that are accessible, regardless of age, gender, socio-economic circumstances, or ethnicity. There is evidence to support community-centred approaches in addressing health equity and to target the deep-rooted health inequalities (8). The public health sector has described need for local governments to provide space for residents to be actively involved in creating and maintaining healthier communities (9).

Through co-development (also known as co-production), an approach of developing products and services through active collaboration (10) allows consumers, providers and experts to work in partnership to deliver optimal outputs. Results from a systematic review which examined community engagement levels illustrated that there is an association between low levels of engagement and poor health outcomes (11). Studies which incorporated high levels of community engagement, through sharing decision-making power and fostering strong professional relationships, experienced a higher degree of success for positive outcomes. A recurring concept was the delivery of the intervention by lay health workers or ‘kin keepers,’ which refers to the teamworking ability and natural contact between women in families. Partnerships such as these may leverage collaborative potential for sustainable transformational change, which will enable continuation of the initiative beyond the pre-defined funding period (12).

A systematic review suggested that the formative research phases [encompassing both the qualitative and quantitative evaluation and their contextual surroundings (13)], are influential in establishing partnerships grounded in trust and vital in sustaining engagement in communities who are sceptical of mainstream health services (14). This understanding guides the definition of the research questions, the outcomes, content of the intervention and mode of delivery. Formative approaches additionally offer opportunities for co-development through community-based participatory research (CBPR) approaches, which involve developing partnerships with community members and key organisations to ensure that they are engaged with public health strategies that may impact them (15).

There is considerable scope for the implementation of health promotion strategies targeted to women from different ethnic backgrounds for NCDs prevention in community settings such as hairdressers and beauty salons. Observational studies have depicted beauty salons as environments where women feel comfortable in exchanging advice, enabled by the relationships held between salon staff and customers and the organisational environment of the salon itself (16). A recent review by Linnan et al. published a literature synthesis of health promotion research taking place in salons and barbershops, combining studies that had been directed to both male and female populations (17). Although extensive and highly detailed, most included studies were published prior to 2013 and were with men. This scoping review provides an opportunity to summarise an updated body of evidence for women.

This scoping review maps and summarises the evidence on public health interventions based in beauty salons for the prevention and management of NCDs in women, with a focus on addressing ethnic inequalities. The themes of interest in the scoped papers were based on intervention design and development, including an exploration of the extent to which formative phases were incorporated into intervention design, including evidence of community participation in intervention development and application of theoretical or conceptual frameworks. Additional areas of focus include methods of invitation, training and incentivisation of salon staff, evaluation methods used to assess impact of the intervention and whether equity factors were addressed in study design.

## Materials and methods

The protocol of this scoping review is adapted from the framework described by Arksey and O’Malley, which suggests progressing through the review in 6 stages, where applicable (18). The methodology also incorporates the recommendations made by Levac et al., and the guidance provided in the resources of the Cochrane Training and the Joanna Briggs Institute (19–21). The Prisma checklist for Scoping Reviews was used as guidance for reporting (22).

### Study eligibility criteria

Intervention studies were comprehensively searched for using the keywords mentioned in Table 1. The searches were conducted across the following electronic databases (PubMed, Web of Science and OVID) on 20th June 2020 and updated on 10th January 2023, with reference lists also screened.

**Table 1:** Keywords and mesh terms utilised within the search strategy.

*Participants, Concept and Context Framework*	*Term*	*Keyword*	*Mesh*
Participants	Ethnically diverse women Ethnic minority	Women Females Black-African Black-Caribbean Mixed ethnicity Indian Pakistani	*(mesh terms not used for this component as too restrictive during searches)*
Concept	Salon-based health intervention	Nail bar Hair salon Hairdressers Intervention	Beauty culture Intervention study Health promotion Health education
Context	NCD prevention	Non-communicable disease	*(mesh terms not used for this component as too restrictive during searches)*

In order to achieve a focussed scoping review and relevant discussion, the ‘PCC’ (participants, concept, context) framework was utilised (21). To be eligible for inclusion, studies had to include salon-based health interventions (concept) focused on NCDs prevention (context) and targeting women from different ethnic backgrounds (participants). In this review, salons refer to establishments where cosmetic, hair and nail treatments are performed by salon staff (or therapists) on clients. We recognise that classifications are context specific and where authors used a specific label we used that in the text (e.g., African American). Studies also had to be published in English. Interventions targeting tanning-related behaviours and interventions focussing on the health of salon staff rather than their clientele were excluded.

The inclusion and exclusion criteria were developed through an iterative process, as suggested by Arksey and O’Malley in their methodological framework, allowing amending and refining the search strategy through understanding of the literature (18). This iterative process enables the authors to ensure that comprehensive terms are used so that all relevant studies are included, which contrasts to systematic reviews where researchers explicitly define the inclusion and exclusion criteria from the outset and the theme underpinning the included papers is relatively narrow (18).

### Study selection

Study selection was split into two stages. Stage 1 involved screening titles and abstracts against the above criteria to identify relevant papers. The second stage involved reading the full text of eligible papers from the first stage.

**Figure 1:**
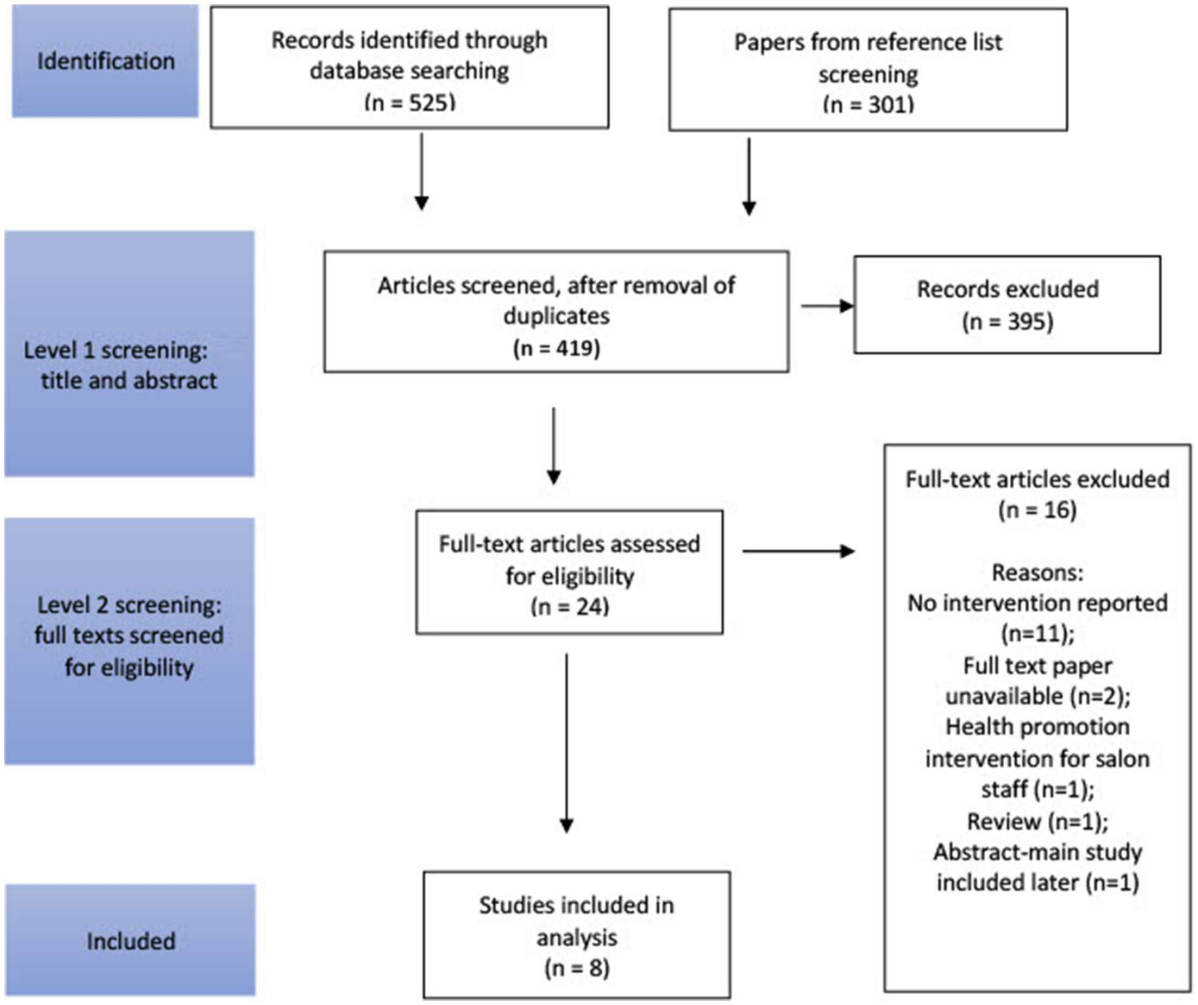
A flowchart to illustrate the selection procedure of the extracted papers, adapted from the PRISMA model (22).

One author (PK) independently screened titles and abstracts identified by the searches. Full-text papers were then retrieved and screened by two authors (PK & MM). Any disagreements were discussed and resolved by consensus; a third reviewer was invited to adjudicate (SH). This process is illustrated by the flowchart in Figure 1, including reasons for exclusion.

### Data extraction

Data were charted iteratively and involved collating the characteristics of each study that are relevant to the scoping review’s research questions and aims (21). This allowed us to create a descriptive summary of aspects such as the contextual background and modes of delivery using a bespoke designed form (19). For this scoping review, a preliminary charting form was designed and pilot-tested on 5 studies, in accordance with the PRISMA guidelines, to confirm that all necessary data were collected (22). Following testing, the form was further modified. The full list of recorded variables can be found in the Results section.

### Approach to evidence synthesis

The final stage of this review involved providing a detailed overview of the included studies to understand how interventions were designed, implemented and evaluated. This was conducted for numeric and thematic synthesis, as recommended by Arksey and O’Malley’s and Levac’s guidance (18, 19).

## Results

### Identification of relevant studies

A total of 419 titles and abstracts were screened to identify studies that were relevant and met the inclusion criteria by the primary investigator. Four papers were considered to be relevant at stage 1 of screening, however we were unable to obtain sufficient detail on these studies on further inquiry. After the removal of duplicates, 24 papers were included in stage two of the screening process, as depicted in Figure 1, whereby the full paper was read and checked against the inclusion and exclusion criteria. Following reference list screening, 8 papers were deemed appropriate for inclusion in the scoping review. Reasons for exclusion included: no intervention was reported (*n* = 11); full text paper was unavailable (*n* = 2); health promotion intervention was intended for salon staff (*n* = 1); review paper (*n* = 1); and abstract for which the main study was included in our later selection (*n* = 1). All studies were conducted in the United States and included NCDs prevention interventions related to cancer (*n* = 4), chronic kidney disease (n = 1), hypertension (*n* = 1), stroke (*n* = 1) and promotion of fruits and vegetable intake (*n* = 1). All studies included African American women and only one study included participants from other African as well as Hispanic ancestries. The number of participants ranged from 12 to 8,148. Four studies did not report information related to participants socioeconomic circumstances. The percentage of participants educated to high school level ranged from 12 to 50%. Additional characteristics of included studies are given in Table 2.

**Table 2:** Brief summary description of scoped studies.

Title, authors, year	Ethnicity and socio-economic circumstances of participants; sample size	Primary objectives	Study design (including whether formative studies were undertaken prior to intervention)	Extent of co-development (e.g., with salon staff, clients, local community organisations, local councils)	Health issue/behaviour of interest	Theoretical or conceptual framework?	Effect on primary outcomes?
Breast cancer screening adherence in African American women: Black cosmetologists promoting health. Sadler et al. (28).	African American, 201 participants. SEC data not reported.	To examine the potential of having therapists acting as lay health educators for breast cancer.	Cluster randomised controlled trial. Therapists randomised into active arm or passive arm; both received one-on-one training and printed materials, but active arm received more in-depth training (i.e., African American ancestral storyteller) and additional materials.	Limited co-development described. Therapists recruited clients.	Breast cancer education to improve knowledge and to increase adherence to screening guidelines.	No framework referred to.	Not reported.
Using Community-Based Participatory Research Methods to Reach Women With Health Messages: Results From the North Carolina BEAUTY and Health Pilot Project. Linnan et al. (24).	111 African American participants, 51 White participants. At baseline, 9.3% had been educated to 11^th^ grade or less; 34.6% were high school graduates; 24.1% had undertaken some college; 32.0% were graduates or postgraduates. 44.3% had a household income of less than 24,999; 32.2% had household incomes of $25–49,999; 23.5% had a household income of $50,000+.	To assess the feasibility of collaborating with beauty salon staff to deliver health promotion messages.	Pre-post-test. Intervention delivered in 2 salons, one serving predominantly African American clientele and the other White clientele. No comparator used. Training involved a four hour workshop conducted by project staff and role-play exercises. Formative work done to examine therapists’ willingness to participate.	Co-development: community-based participatory research approach; project’s advisory board included licenced therapists, beauty school directors, beauty product distributor, a local health department representative and local community residents. Pilot study.	Healthy eating, exercise and using the cancer information services (via a hotline)	No framework referred to.	Immediately post intervention: 98.2% of African American participants and 96.4% noticed the BEAUTY Project education health display; 83.3% of African American participants and 89.3% of White participants spoke to their cosmetologist about the BEAUTY Project during their appointment; 71.2% of African American participants and 53.8% of White participants discussed the cancer prevention health brochures with their cosmetologist. 12 months post-intervention from a convenience sample of 56 participants (39 African American, 17 White): 60.7% reported an ‘increased readiness’ to change at least one health behaviour following the BEAUTY Project; 55% reported they had made changes to their health behaviours; of this 55, 74.1% reported calling the cancer hotline, 70.9% reported exercising for at least 30 min a day, 70.9% ate at least 5 fruit and vegetables every day since participating in the Project. No evidence of ‘improvement’ provided -no baseline data reported.
Healthy hair starts with a healthy body: hair stylists as lay health advisors to prevent chronic kidney disease Madigan et al. (29).	African American, 8,148 participants. SEC data not reported.	To promote health behaviours and increase risk awareness of diabetes and hypertension, in relation to chronic kidney disease.	Cluster randomised controlled trial. Training to therapists through two four hour workshops and provided with information materials.	Co-development of training curriculum with physicians and nutritionists from local community.	Hypertension and diabetes education regarding lifestyle behaviours and risk awareness.	No framework referred to.	Of those who ‘wished to make changes’ (figure not reported: Healthy eating – 46% Increase in exercise – 32% Differences in outcomes between intervention and control not reported on.
Hair salon stylists as breast cancer prevention lay health advisors for African American and Afro-Caribbean women. Wilson et al. (25).	Predominantly African American and Afro-Caribbean -1,185 participants: 92% of African ancestry, 7% of Hispanic ancestry. SEC data not reported.	To assess the effectiveness of breast health promoting messages administered by salon stylists.	Cluster randomised control trial. Salons were randomly assigned to either the experimental arm (active health promotion regarding breast cancer) or the control group (28 vs. 12 respectively). Training for stylists co-developed and in the form of two, two-hour workshops, a reference handbook and ongoing support.	Co-development with partners from Arthur Ashe Institute, faculty at the State University of New York Downstate Medical Centre and the ‘Health and Beauty Council’ (included local community health leaders, breast cancer survivors and academic partners). Based on previous preliminary studies.	Breast cancer knowledge and examination behaviours.	Social Cognitive Theory	Breast self-examination in last 3 months – OR: 1.60; 95% CI: 1.20–2.13. Received a clinical breast exam – OR: 1.20; 95% CI: 0.94–1.52. Intends to have a clinical breast exam in the next year – OR: 1.87; 95% CI: 1.11–3.13. Received a mammogram in the last 3 months – OR: 1.21; 95% CI: 0.84–1.76. Intends to have mammogram in the next year – OR: 1.34; 95% CI: 0.88–2.04.
The Challenges of Community-Based Research: The Beauty Shop Stroke Education Project. D. Kleindorfer et al. (26).	African American, 383 participants. 31.1% educated to high school and 68.9% had partially undertaken or completed a college degree.	To demonstrate sustained improvement in knowledge of stroke warning signs by educating women in black beauty shops.	Systematic case study. No comparator group. Beauticians received training at a ‘Beauty Shop Training Luncheon’, where the PI presented information on stroke warning signs and risk factors, a stroke survivor discussed her experiences, and beauticians were surveyed pre- and post-educational session on their knowledge.	Limited co-development described – community contacts used to invite beauticians. No piloting work mentioned.	Education on stroke.	Health Belief Model and Social Cognitive Theory	Statistically significant improvement in knowledge of three warning signs. Statistically insignificant improvement in knowledge of three risk factors. Knowledge improvement warning signs – baseline: 40.7%; 6-week survey: 50.8%; 5 months: 50.6% (*p* = 0.006). Knowledge improvement risk factors – baseline: 46.7%; 6-week survey: 54.0%; 5 months: 48.7% (*p* = 0.53).
Beauty Salon Health Intervention Increases Fruit and Vegetable Consumption in African American Women. Johnson et al. (30)	African American, 20 participants. 50% of the experimental group had completed high school and 20% had bachelor’s degrees. In the comparator group, 60% had completed high school and the proportion of those with bachelor’s degrees were not reported.	To assess if a beauty salon-based health intervention will lead to increased fruit and vegetable consumption.	Quasi-experimental study. Participants in one salon were in the intervention arm, participants in the other were in the control arm. Training provided to therapists, which included how to implement the motivational sessions and practise sessions with feedback. Formative work by pilot testing survey for readability.	No co-development methods described. Preliminary work, prior to intervention development, involved screening of the treatment beauty salon. The pre-test questionnaire pilot tested for readability.	Healthier eating, active lifestyles and water consumption knowledge and uptake.	No framework referred to.	Increase in fruit and vegetable consumption – Mean [SD] comparison group: pre-test = 3.8 [1.8], post-test = 3.5 [1.3]; treatment group: pre-test – 1.8 [1.0], post-test = 3.4 [1.3]. Increase in physical activity: comparison group: pre-test = 24.9 [17.2], post-test = 33.5 [26.8]; treatment group: pre-test – 29.0 [17.4], post-test = 32.5 [10.3]. Increase in water consumption: comparison group: pre-test = 4.0 [2.9], post-test = 4.6 [2.9]; treatment group: pre-test – 4.9 [2.8], post-test = 6.8 [2.2].
A Cluster Randomised Controlled Trial to Increase Breast Cancer Screening Amongst African American Women: The Black Cosmetologists Promoting Health Programme. Sadler et al. (23)	African American; 984 participants. At baseline: 12% had been educated to high school; 52% had undertaken some college; 34% had completed college.	Improved adherence to mammography screening guidelines.	Cluster randomised control trial (RCT); salons were randomly assigned to either the experimental arm (breast cancer intervention arm) or the control group (diabetes intervention). Therapists given training by African American ancestral storytellers, and were given hands-on training materials every two weeks	Co-development with local church women leaders, who invited therapists. Based on previous piloting and feasibility research.	Breast Cancer screening guideline adherence.	Health Belief Model	Newly adherent mammography screening: two times higher than the women in the diabetes control group OR 2.0 (95% CI: 1.03–3.85).
Implementation of a community-based outreach hypertension programme in an urban beauty salon. Shennen Smith (27).	African American, 12 participants. SEC data not reported.	To determine if the outreach program will increase the participants’ knowledge of hypertension.	Systematic case study. No comparator group. Training provided to nursing assistants acting as medical volunteers to share information materials, and use data collection documentation and equipment.	Limited co-development described – participants recruited by the therapists working at the salon. Pilot study.	Hypertension education on lifestyle behaviours and management techniques.	Chronic Care Model	Hypertension Evaluation of Lifestyle and Management Scale – Z score: −0.106 Hypertension Self-Care Profile Scale – Z score: 1.270.

### Community participation

Evidence of co-development with key stakeholders or experts from within the community was exhibited in three of the eight papers (23–25). Linnan et al.’s intervention included the establishment of a ‘Beauty advisory board’, consisting of beauty school directors, therapists, a beauty product distributor, a local health department representative, local community residents, a community outreach worker from the Cancer Information Service and members of the research team. A beauty school director provided a list of local licenced therapists and salon owners that were involved in reviewing the survey questions, the analysis of data, and appraising training materials for the therapists (24). Key stakeholders were also invited to be involved in Wilson’s investigation, in the form of the Health and Beauty Council, comprising of salon owners, breast cancer survivors, and leaders of local media organisations (25). Collaborative efforts between the stakeholders enabled the development of the training workshops and evaluation questionnaires, definition of how results would be collected and assessment of results. Sadler’s study (23) partially addressed co-development, as the intervention was an extension of a church-based community-campus research partnership. African American church leaders recognised that church-based programmes focussing on breast cancer were not reaching new audiences, and were responsible for inviting therapists and in reviewing the programme details.

### Theoretical and conceptual frameworks

The Health Belief Model (HBM) was referred to in two studies (23, 26), the Social Cognitive Theory (SCT) in two studies (25, 26) and the Chronic Care Model (CCM) in one study (27). Perceived susceptibility, severity, benefits, and barriers, and cues to action served as the foundation of Sadler et al.’s (23) intervention. Pre- and post-intervention surveys were conducted to evaluate each participants’ understanding of their susceptibility to breast cancer, their knowledge of it being a serious health issue, what measures might be taken to protect themselves from the disease, and current adherence to recommended advice regarding screening.

In the Beauty Shop Stroke Education Project, the authors discussed the role of the HBM in regards to the importance of improving risk factors, by emphasising a person’s vulnerability to stroke and thus compelling appropriate behaviour change to reduce both susceptibility and severity of illness. This initiative also made reference to the SCT, and thus specified a key objective of achieving high participation rates by African-American women in the community to facilitate and sustain extensive engagement from other women of this ethnicity (26).

SCT was used as a framework for the development of the content of training and the health promoting messages delivered in Wilson et al.’s intervention. However, no examples of the key messages received by clients were reported, making it unclear how exactly the SCT informed the content of the scheme. However, given that outcome measures included completing or intending to engage in a breast self-examination, clinical breast examination and mammogram, it is plausible that these messages explained details of a breast self-examination (relating to the ‘behavioural capability’ and ‘modelling’ constructs), as well as the significance of doing so (relating to the ‘expectations’ construct) (25).

The CCM stipulates that proactive changes in primary care are needed to better support those who have chronic conditions, and formed the basis of Smith’s community-based hypertension programme. Nursing assistants were trained as volunteers and an on-site nurse and health care provider were made available at each session of this intervention to introduce the concept of delivery system design, and the establishment of self-care goals during the educational sessions incorporated the principle of self-management. Additionally, participants were referred to additional resources to further their knowledge of hypertension risk factors and to use the self-monitoring log to enter their data, thus using the clinical information systems aspect of the model (27).

### Training of lay health educators

In 7 out of 8 studies, salon therapists were offered training. Training duration varied from “brief presentations” during a “Beauty Shop Training Luncheon” to 8 h (2 × 4 h) training workshops. Where training was provided to salon therapists, most of the time the training was delivered by the research team (6 out of 7 studies) and included practical discussion on how salon therapists should engage in conversations with their clients (6 out of 7 studies). Sadler et al. (28) provided one-on-one training delivered by the principal investigator supported by additional guidance from an African-American ancestral storyteller and educational resources. In Sadler et al. (23) hands-on training materials were delivered every two weeks which to further support salon therapists. Linnan et al. described training workshops which were also conducted by members of the research team and included role play exercises (24). Wilson et al. reported training was also delivered by the research team and included educational resources as well as ongoing support to therapists (25). Madigan et al. reported training was delivered by experts recruited from each local community (e.g., physicians and nutritionists). One-on-one practises and monitoring by research staff were also offered (29).

Incentives for participation in the training/study included the provision of once or monthly financial payments to salon therapists (10 to 50$ to compensate for their time and/or travelling expenses), professional development opportunities valued at 800$ and 4$ to 5$ discount vouchers passed on to clients. Wilson et al. highlighted that although financial incentives were provided as well as transportation to pick up/drop off salon therapists for training, only one third of therapists completed the training (25). Linnan et al. also discussed that impact of financial incentives should be further explored (24).

### Evaluation

Outcome evaluation was conducted for all of the eight interventions. Frequently appearing outcome indicators were retention of knowledge (23, 24, 26–28), intention to change behaviour (24, 25, 29), changes in behaviour (23–25, 28–30), and changes in risk awareness (23, 26, 27, 29). Kleindorfer and Madigan took similar approaches to outcome evaluation, in that surveys were administered both after a short post-intervention period (six weeks and immediately after the intervention, respectively) and after a longer duration (five months and six months, respectively), enabling the research teams to understand how effective their mode of intervention deliveries were in facilitating retention. The overall results are indicative of positive changes occurring through hosting a health promotion intervention in a beauty salon, however these were not always statistically significant.

Five of the interventions incorporated process evaluation (23–25, 27, 28). Indicators included attrition rate (23, 27), therapists’ insights of the intervention (24, 28), clients’ observations from the intervention (24), confidence of clients in their therapists to deliver the correct health messages (23) and the confidence and willingness of therapists to deliver health promotion messages (25). There was heterogeneity in how each of these was assessed. When assessed, clients displayed confidence and acceptability in their therapists as lay health educators, and therapists noted that clients were actively interested in receiving the specified health messages (23, 28).

Table 3 reviews the outcome and process evaluation results by using dimensions of the RE-AIM framework (reach, effectiveness, adoption, implementation and maintenance) (31), with less than one in four studies reporting aspects related to adoption, implementation and maintenance. An important aspect that arose in Madigan et al.’s intervention was that some beauty therapists did not complete training, despite incentivisation, suggesting implementation issues. It was also reported that financial difficulties and business closures meant that some sites had to stop abruptly, although no formal data were collected on the closures.

**Table 3:** Study results within context of the RE-AIM framework (31).

Dimensions	Percentage of studies that reported upon each dimension
*Reach*	
Description of target population (clients)	100
Description of target population by SEC (clients)	50
Response and attrition rates of clients	100
Response and attrition rates of clients by SEC	12.5
*Effectiveness*	
Primary outcome evaluation	75
Use of qualitative methods to understand outcomes	37.5
*Adoption*	
Response rates of salons invited	25
Socio-demographics of staff delivering the intervention	12.5
Response rates of salon staff invited	25
Cultural adaptation of intervention	25
Co-development with salon staff and clients	37.5
*Implementation*	
Measures taken to aid fidelity of lay health educator training	25
Measures taken to aid fidelity of therapist to protocol	12.5
Incentives offered to salon/staff	87.5
Incentives offered to participants	50
*Maintenance*	
Measure of primary outcome at >6 months follow-up after final intervention contact	25
Attrition rate of participant study completion	25

Socio-economic circumstances were partially measured in some of the studies and highest level of education and household income were reported by the participants in Wilson et al.’s (25) and Linnan et al.’s (24) investigations at baseline, however their role in equity of coverage or influence on outcome achievement and participant attrition was not described.

## Discussion

This scoping review found that interventions delivered in beauty and hairdressing salons for the prevention and management of NCDs in women from different ethnic backgrounds can leverage collaborative potential for sustainable transformational change, and provide novel opportunities to address health equity. However the utility of formative research in the scoped studies was weakly reported and community participation varied considerably. Theoretical and conceptual frameworks were not consistently used, and there was inadequate process evaluation to ensure equitable reach and retention of targeted groups across socioeconomic circumstances.

### Community participation

Involving the community in the research process includes several sub-genres (e.g., integrated knowledge translation, community-based participatory research, co-production) that share similar views in relation to the importance of building authentic partnerships and valuing different forms of knowledge, including lived experience, but vary in the extent to which they aim to promote emancipation, power and capacity of knowledge users. Community based interventions can empower communities via the process of co-development and capability building and also through strengthening community capital of communities. However, most of these studies did not measure acceptability of feasibility of the interventions or promote long-term sustainability, so it is difficult to say that whether hairdressers and salon staff were truly ‘empowered.’

Our review highlighted that community participation was implicit in each of the scoped studies, as the interventions required partnerships and professional relationships to be formed to enable successful programme implementation, but were only explicitly acknowledged by less than half of the scoped studies (23–25, 27). The extent of community participation also varied, with few interventions employing participatory methodologies from the outset. Community participation has been argued to be a central component of interventions seeking to deliver a positive public health purpose in an equitable manner (14). Public Health England have recognised the importance of community participation in reducing health inequalities, particularly in increasing capacity and developing strong collaborative partnerships by creating roles such as community champions and peer-to-peer supporters in place-based approaches (32). Promoting community participation entails developing a ‘bottom up’ health promotion strategy, whereby residents, commissioners and health service providers collaborate in using community assets to bring about wellbeing (32). It is often argued that participatory approaches only consider the ‘surface’ level of cultures, thus allowing only for cultural sensitivity as opposed to fundamental shifts in community-based agency (33). Considering that the appraised studies targeted women from different ethnic backgrounds, with retention, where reported, being considered poor (23), suggests the need to pay more attention to the principle of equity and employ more ‘deep level’ strategies that address the broader social and cultural context of participants, particularly when there was limited consideration of how SEC (socio-economic circumstances) may influence intervention delivery, participation, and achievement of desired outcomes, which may hinder long-term sustainability of the intervention within targeted vulnerable communities who are most in need of such services.

### Theoretical and conceptual models

SCT and HBM were referred to in two studies each, and one paper based the intervention on the CCM. Delivering training sessions that are informed by the SCT may increase an intervention’s effectiveness, as approached by Wilson et al., as attention is given to enhancing the capabilities of community members to support each other, through strengthening communication skills, furthering awareness on practical issues that may serve as barriers, and improving receptivity to this form of information (34). This closely resembles the principles behind community-based health care interventions. By aiding understanding of how susceptibility can arise, preventive measures and signposting to relevant resources and services, participants of interventions informed by the HBM may be further empowered to incorporate feasible recommendations into their lifestyles, again resonating with the principles of valuing autonomy in community-level health interventions (35). Of note was the finding following Sadler et al.’s intervention that a smaller proportion of women perceived breast cancer as a major health problem, at follow-up, indicating that they felt better equipped to reduce this health threat after receiving relevant advice (23).

The CCM shifts focus away from the individual and considers the roles of the community and health care system to examine how they can be targeted to improve community health (36). This supports Bandura’s findings that a multifaceted approach is required for the purposes of disease prevention and management, including adaptations to how health service systems function (34). In this regard, Smith sought to create partnerships between community organisations (beauty salons) and primary health services to improve hypertension self-management practices (27).

A lack of commitment to theories underpinning community-level health intervention has been suggested as a core reason behind the often-observed inconsistency in results (37), with contention that integration of theoretical frameworks are necessary for achieving health-related outcomes (38). However, it has also been acknowledged that there is disconnection between each of the many theoretical frameworks despite their aim of supporting health promotion, and this serves to hinder public health strategies (39). There is argument to shift towards an overarching theoretical framework that is comprising of recurring tenets of separate theories to accelerate improvements.

### Training of lay health educators and incentives

Training of lay health educators in delivery of the interventions was present in almost all the interventions. The inclusion of guidance, practise sessions and hands-on educational resources can enhance salon therapists’ capability and confidence in disseminating key health-related messages. In most studies, training was delivered by the research team. Incentivisation of therapists was employed to facilitate engagement with initiatives, and to ensure training protocols were completed. However, it was unclear whether those were appropriate to enable salon therapists’ participation. Sustainability of these programmes was also infrequently considered, particularly in consideration to financial and time constraints. Fostering collaborations with local healthcare teams, for example, could contribute to the potential scalability and sustainability of health interventions.

### Evaluation

Guidelines for complex intervention development and evaluation reinforce the importance of conducting process evaluations to better understand why and how an intervention is achieving (or not) the expected outcomes (40). The RE-AIM framework was developed to understand how interventions are adopted, implemented, and sustained over time. Integrating iterative cycles of reflection and action throughout intervention implementation and maintenance also enables a timely evaluation of elements of the RE-AIM framework and the identification of gaps and opportunities to optimise the public health impact of interventions (41), however these measures were generally poorly reported on, limiting our understanding of real-world impact of interventions.

Understanding representativeness in terms of cultural and socio-economic circumstances and characteristics is a key element of the RE-AIM framework as they contribute to health equity (42). Similarly, SEC may also influence adoption, implementation, effectiveness, and maintenance, which should be considerations for future studies. To achieve sustained improvement public health commissioners should seek to understand the ‘deep structures’ operating within a community, such as their values, perceptions, social relationships and contexts, by taking a culture-centred approach (CCA) (33). Alongside intersecting with the socioecological framework, this enforces community empowerment, a concept which Wallerstein et al. describe as directly corresponding with the notion of culture-centredness, and when combined, facilitate adaptiveness of the intervention, community agency, and sustained improvement in outcomes. All three of these principles are crucial to achieving health equity.

### Strengths and limitations

This rigorously conducted scoping review identified a gap in the literature, and aligned with well recognised review standards (18, 20, 21). Our review systematically and comprehensively extracted papers and mapped the relevant evidence, discovered consistent findings and incorporated the RE-AIM framework to understand dimensions of equity when evaluating research processes and outcomes (31, 43, 44). Limitations include a relatively small number of studies with heterogenous methods and outcomes and limited follow up periods to explore sustainability. Additionally, scoped studies predominantly targeted African-American women and were all carried out in the United States, making it challenging to draw conclusions about the effectiveness and potential of salon-based health promotion interventions across the multi-ethnic spectrum for women globally.

### Future directions

Future interventions could be strengthened by underpinning of theoretical or conceptual frameworks, which may facilitate the delivery of key health messages which closely resonate with the manner in which the targeted community perceive their own health and health risks; this is in following from evidence which indicates that interventions applying a theoretical basis deliver a greater degree of success.

A consistent integration of formative phases of research, often a crucial factor in the sustainability of such interventions through developing partnerships based on authenticity (14) is an important consideration. These approaches may also positively influence equity, as interventions may be targeted to underserved communities. However, it is imperative that the intervention reaches and retains engagement with individuals from the least privileged SEC positions. In this regard, outcome and process evaluation should be conducted with consideration to SEC characteristics of the participants, to enable consideration of how further effectiveness can be achieved. Process evaluation should also be conducted to ascertain the cost-effectiveness of future interventions to allow comparisons to be made with other potential strategies for similar outcomes. This will provide evidence for the adoption and maintenance dimensions of the RE-AIM framework, as well as aiding sustainability of such ventures.

## Conclusion

Our evidence synthesis concerning the use of salon-based health promotion interventions relating to NCDs and delivered to women from different ethnic backgrounds, conveys the scarce and yet heterogenous field of research and the myriad of components which are involved in the design, implementation and evaluation. Participatory approaches, such as CBPR, although perceived as a valuable methodology to intervention development, effectiveness and sustainment were not frequently reported. Application of theoretical and conceptual frameworks was less prevalent amongst the scoped studies as well; however, it was apparent that there is considerable intersection between the objectives of each intervention and the core tenets of the theories described within this review. Similarly, insufficient process evaluation was identified, which may hinder timely inferences about the potential for reach and adoption, especially as although vulnerable communities were targeted, where reported there was high loss to follow-up. Future salon-based interventions targeting NCD management and prevention in women from different ethnic backgrounds should explore how to better strengthen community-based partnerships, particularly through training programmes for the salon therapists.

## Author contributions

PK (early career researcher), MM, and SH formulated the research question and analytical plan and completed the analyses. All authors contributed to the article and approved the submitted version.

## Funding

This work is supported by a grant held by MM and SH from the National Institute of Health Research (NIHR) Research for Patient Benefit (RfPB) Programme (NIHR202769): Hairdressing salons to promote NHS online application to reduce under-diagnosis of cardiovascular risk factors among women in London’s deprived and ethnically-diverse neighbourhoods: a feasibility study. SH is funded by the Medical Research Council (MRC): MR/N015959/1, MR/S009035/1, MR/R022739/1, MR/S003444/1, MR/Y009983/1, and MR/X003078/1.

## Conflict of interest

The authors declare that the research was conducted in the absence of any commercial or financial relationships that could be construed as a potential conflict of interest.

## Publisher’s note

All claims expressed in this article are solely those of the authors and do not necessarily represent those of their affiliated organizations, or those of the publisher, the editors and the reviewers. Any product that may be evaluated in this article, or claim that may be made by its manufacturer, is not guaranteed or endorsed by the publisher.
