# Corrigendum: A scoping review of the evidence available for the use of salons as health promotion environments, for the prevention and management of non-communicable diseases in women from different ethnic backgrounds

**DOI:** 10.3389/fpubh.2023.1303052

**Published:** 2023-11-20

**Authors:** Prerana Kaneri, Marjorie Lima do Vale, Seeromanie Harding, Mariam Molokhia

**Affiliations:** Department of Population Health Sciences, School of Life Course and Population Sciences, King's College London, London, United Kingdom

**Keywords:** salon, ethnicity, community partnership model, health inequalities, review

In the published article, there was an omission in the Funding statement.

The funding statement for the Key Development Project of the Department of Science and Technology was displayed as

“Funding: This work is supported by a grant held by Dr. Mariam Molokhia & Professor Seeromanie Harding from the National Institute of Health Research (NIHR) Research for Patient Benefit (RfPB) Programme (NIHR202769): Hairdressing salons to promote NHS online application to reduce under-diagnosis of cardiovascular risk factors among women in London's deprived and ethnically-diverse neighbourhoods: a feasibility study.”

The correct Funding statement appears below.

